# Suppressed Vascular Leakage and Myocardial Edema Improve Outcome From Myocardial Infarction

**DOI:** 10.3389/fphys.2020.00763

**Published:** 2020-07-09

**Authors:** Xiujuan Li, Björn Redfors, Miguel Sáinz-Jaspeado, Shujing Shi, Pernilla Martinsson, Narendra Padhan, Margareta Scharin Täng, Jan Borén, Malin Levin, Lena Claesson-Welsh

**Affiliations:** ^1^Cyrus Tang Hematology Center, Collaborative Innovation Center of Hematology, State Key Laboratory of Radiation Medicine and Protection, Soochow University, Suzhou, China; ^2^Beijer and Science for Life Laboratories, Department of Immunology, Genetics and Pathology, Rudbeck Laboratory, Uppsala University, Uppsala, Sweden; ^3^Department of Molecular and Clinical Medicine / Wallenberg Laboratory, Institute of Medicine, The Sahlgrenska Academy at University of Gothenburg and Sahlgrenska University Hospital, Gothenburg, Sweden

**Keywords:** VEGF, VE-cadherin, adherens junctions, endothelial, vascular permeability, myocardial infarction

## Abstract

**Aim:**

The acute phase of myocardial infarction (MI) is accompanied by edema contributing to tissue damage and disease outcome. Here, we aimed to identify the mechanism whereby vascular endothelial growth factor (VEGF)-A induces myocardial edema in the acute phase of MI to eventually promote development of therapeutics to specifically suppress VEGFA-regulated vascular permeability while preserving collateral vessel formation.

**Methods and Results:**

VEGFA regulates vascular permeability and edema by activation of VEGF receptor-2 (VEGFR2), leading to induction of several signaling pathways including the cytoplasmic tyrosine kinase c-Src. The activated c-Src in turn phosphorylates vascular endothelial (VE)-cadherin, leading to dissociation of endothelial adherens junctions. A particular tyrosine at position 949 in mouse VEGFR2 has been shown to be required for activation of c-Src. Wild-type mice and mice with phenylalanine replacing tyrosine (Y) 949 in VEGFR2 (*Vegfr2*^*Y*949*F*/*Y*949*F*^) were challenged with MI through permanent ligation of the left anterior descending coronary artery. The infarct size was similar in wild-type and mutant mice, but left ventricular wall edema and fibrinogen deposition, indicative of vascular leakage, were reduced in the *Vegfr2*^*Y*949*F*/*Y*949*F*^ strain. When challenged with large infarcts, the *Vegfr2*^*Y*949*F*/*Y*949*F*^ mice survived significantly better than the wild-type strain. Moreover, neutrophil infiltration and levels of myeloperoxidase were low in the infarcted *Vegfr2*^*Y*949*F*/*Y*949*F*^ hearts, correlating with improved survival. *In vivo* tyrosine phosphorylation of VE-cadherin at Y685, implicated in regulation of vascular permeability, was induced by circulating VEGFA in the wild-type but remained at baseline levels in the *Vegfr2*^*Y*949*F*/*Y*949*F*^ hearts.

**Conclusion:**

Suppression of VEGFA/VEGFR2-regulated vascular permeability leads to diminished edema without affecting vascular density correlating with improved myocardial parameters and survival after MI.

## Introduction

Vascular endothelial growth factor-A (VEGFA) plays a protective role in ischemic heart disease and myocardial infarction (MI) by inducing angiogenesis (Kajdaniuk et al., 2011). However, during the early phase of coronary artery occlusion, VEGFA induced by the acute tissue hypoxia and ensuing inflammation promotes enhanced vascular permeability and extravasation of plasma constituents, causing edema, eventually resulting in tissue damage, and inflammation (Garcia et al., 2019; Hausenloy et al., 2019).

Vascular endothelial growth factor-A together with VEGFB, VEGFC, VEGFD, and placenta growth factor (PlGF) form a family of structurally related growth factors, which bind to three related receptor tyrosine kinases; VEGF receptor-1 (VEGFR1), VEGFR2, and VEGFR3 (Simons et al., 2016). While VEGFR1 is broadly expressed, VEGFR2 and VEGFR3 are mainly although not exclusively expressed on blood vascular and lymphatic endothelial cells (ECs). VEGFA is a potent inducer of vascular permeability; in accordance, it was originally identified as vascular permeability factor (VPF) (Dvorak, 2002). Binding of VEGFA to VEGFR2 leads to activation of the receptor tyrosine kinase and phosphorylation of tyrosine residues (Y) 949, 1052, 1057, 1173, and 1212 (mouse sequence numbering) in the receptor intracellular domain (Matsumoto et al., 2005). Phosphorylated (p) Y949 in VEGFR2 mediates c-Src activation at EC junctions (Sun et al., 2012; Li et al., 2016), and loss of pY949 signaling in *Vegfr2*^*Y*949*F*/*Y*949*F*^ mice results in reduced vascular leakage in response to VEGFA administration, in the dermal and tracheal vasculature (Li et al., 2016). Moreover, vascular permeability and edema are lower in *Vegfr2*^*Y*949*F*/*Y*949*F*^ mice compared to wild-type, after challenge with GL261 glioma, B16F10 melanoma and RipTag neuroendocrine cancer. The enforced vascular barrier in the *Vegfr2*^*Y*949*F*/*Y*949*F*^ mice correlates with decreased metastatic spread from melanoma and neuroendocrine cancer in mice (Li et al., 2016).

Several therapeutic strategies are available to suppress VEGFA biology including neutralizing antibodies Avastin/Lucentis, the recombinant VEGF receptor fragment Aflibercept and a broad range of small molecular weight tyrosine kinase inhibitors. These drugs are used to treat conditions associated with exaggerated formation of dysfunctional, leaky vessels such as diabetic retinopathy and age-related macular degeneration (Avastin/Lucentis, Eylea) (Ferrara and Adamis, 2016) and in treatment of highly vascularized solid tumors such as renal cell carcinoma, hepatocellular carcinoma, colorectal cancer (VEGFR2-targeting kinase inhibitors) (Bhullar et al., 2018). However, complete suppression of VEGFA/VEGFR2 in disease may lead to side effects such as geographical atrophy in retinopathy and exacerbation of the disease in cancer (Ebos et al., 2009; Paez-Ribes et al., 2009). It is therefore important to understand how VEGFA/VEGFR2 contributes to disease and to inhibit only those aspects of the biology that contributes to disease such as induction of vascular permeability, while sparing other aspects such as angiogenesis. This is particularly relevant in myocardial insult where VEGFA biology plays very different roles during different stages of the disease.

Vascular endothelial growth factor-A-induced vascular permeability requires disintegration of homophilic interactions between vascular endothelial (VE)-cadherin molecules in adherens junctions (Dejana et al., 2008), in prevenular capillaries and postcapillary venules (Honkura et al., 2018). In these vascular beds, VE-cadherin is phosphorylated constitutively on Y658 and Y685, through flow-dependent activation of c-Src (Orsenigo et al., 2012). Acute stimulation with VEGFA further increases VE-cadherin phosphorylation (Li et al., 2016). Phosphorylation on Y685 in VE-cadherin correlates with elevated vascular permeability (Orsenigo et al., 2012; Wessel et al., 2014).

Here, we show that genetic suppression of VEGFA-induced vascular permeability in conjunction with MI is accompanied by reduced left ventricular wall edema and improved performance in a range of cardiac parameters. The underlying mechanism involves reduced phosphorylation on Y685 in VE-cadherin due to loss in signaling downstream of Y949 in VEGFR2. These data emphasize the detrimental effect of excess vascular permeability and edema in the acute phase of MI.

## Materials and Methods

### VEGFR2Y949F Mouse Model

A mouse model on the C57BL/6J background with knock-in of phenylalanine (F) to replace the tyrosine (Y) at position 949 of VEGFR2 (designated *Vegfr2*^*Y*949*F*/*Y*949*F*^) has been described (Li et al., 2016). Age-matched, (approximately 12 weeks-old) male *Vegfr2*^*Y*949*F*/*Y*949*F*^ mutant and wild-type (WT) littermates from Y949F heterozygous breeding were compared.

### Ethics Statement

All animal studies were approved by the Uppsala University (approval reference number 5.8.18-06789/2018) and Göteborg University Animal Ethics Committees and conform to the guidelines from Directive 2010/63/EU of the European Parliament on the protection of animals used for scientific purposes. Mice were anesthetized using isoflurane and at the end of experiments, sacrificed by cervical dislocation. Care was taken to avoid unnecessary suffering of the animals during the procedure. No animals were excluded from analysis.

### MI Induction

Myocardial infarction was induced in a genotype-blinded manner by left anterior descending (LAD) coronary artery ligation immediately distal to the bifurcation of the left coronary artery (for induction of large infarctions) and at ∼3 mm distal to the bifurcation of the left coronary artery (for induction of smaller infarctions) as described in detail previously (Andersson et al., 2015). To keep the mice sedated and support breathing during the operation, the mice were anesthetized with isoflurane (Forene^®^, AbbVie Inc., North Chicago, IL, United States), orally incubated and connected to a respirator (SAR-830 small animal ventilator, GENEQ) distributing a mixture of oxygen, air and 2–3% isoflurane.

Induction of MI was immediately verified by characteristic changes in the electrocardiographic pattern and akinesis of the left ventricular anterior wall. After verification of infarction, the lungs were hyperinflated, positive end-expiratory pressure was applied, and the chest was closed. The mice received Temgesic (3 mg/ml; 0.1 mL administered intraperitoneally (i.p.) to relieve post-operative pain and recovered spontaneously when the isoflurane was turned off. In a survival study, large infarctions were induced by ligation at ∼1 mm below the atrial appendage, and survival rate was analyzed 24 h after infarction.

At 3 days post smaller infarction, infarcted hearts were collected and snap-frozen in dry-ice/isopentane. Heart lysate from the lower half of the heart (about 5 mm) were analyzed using a multiplex human inflammatory protein biomarker panel (Olink Bioscience; see www.olink.com/content/uploads/2015/12/0696-v1.3-Proseek-Multiplex-CVD-I-Validation-Data_final.pdf). where reactivity depended on detected by two antibodies against each marker. An equivalent anti-mouse panel was not available.

### Echocardiography

Mice were anesthetized with 1,2% isoflurane (Forene^®^, AbbVie Inc., North Chicago, IL, United States), and underwent echocardiography examination at baseline and at 24 h after MI induction, using a VEVO 770 system (VisualSonics, Ontario, ON, Canada), as previously described (Drevinge et al., 2016). The animals’ chests were shaved and hair removal gel was applied to minimize resistance to ultrasonic beam transmission. The mice were then placed on a heating pad and paws were connected to electrocardiographic (ECG) electrodes. A 45 MHz linear transducer (RMV 704) was used for imaging. An optimal parasternal long axis (LAX) cine loop (i.e., visualization of both the mitral and aortic valves, and maximum distance between the aortic valve and the cardiac apex) of >1000 frames/s was acquired using the ECG-gated kilohertz visualization technique. Parasternal short axis cine-loops were acquired at 1, 3, and 5 mm below the mitral annulus. End-diastolic and end-systolic LV volumes and ejection fraction (EF) were calculated by biplane Simpson’s formula using the three parasternal short-axis views and the parasternal long-axis view. M-mode measurements were performed (in the 3 mm level) using the leading-edge method. End-diastole was defined at the onset of the QRS complex, and end-systole was defined as the time of peak inward motion of the interventricular septum. At least three beats were averaged for each measurement. Infarct size was assessed based on wall motion score index (WMSI). WMSI was analyzed by a 24-segments model on the long axis view, three short axis images and 4-chamber views, with the following settings: 0 for normal, 0.5 for reduced wall thickening and excursion in a segment, and 1 for no wall thickening and excursion in a segment. WMSI was calculated as the sum of scores divided by the total number of segments. The stored data was evaluated offline in a genotype-blinded fashion with VevoLab software (VisualSonics).

### Antibodies

The following antibodies were used in immunostaining: rat anti-mouse CD31 (BD Biosciences), goat anti-mouse CD45 (BD Pharmingen), goat anti-mouse fibrinogen (Nordic Immunological Laboratories), rat anti-mouse Ly6G (BD Pharmingen). Fluorescently labeled secondary antibodies were derived from donkey (Invitrogen) or goat (Jackson ImmunoResearch). The following antibodies were used in immunoblotting: goat anti-mouse VEGFR2 (R&D), phospho-VEGFR2 (pY1175) (Cell Signaling), goat anti-mouse VE-cadherin (R&D), anti-GAPDH mouse monoclonal antibody (Millipore). Rabbit antibodies against c-Src (32G6), pY418 Src (D49G4), extracellular regulated kinase (Erk)1/2, pT202Y204 Erk1/2 (197G2), Akt, pT308 Akt were all from Cell Signaling. Anti-pY685 VE-cadherin was kindly provided by Dr. Elisabetta Dejana Uppsala University/IFOM Milano (Orsenigo et al., 2012).

### Immunostaining

Heart cryo-sections (10 μm) at 5 mm from the apex were collected for immunostaining. The sections were fixed in 4% paraformaldehyde for 10 min, washed with PBS, and blocked/permeabilized with 3% BSA/0.2% Triton X-100 in PBS, followed by incubation with primary antibody at 4°C overnight and with second antibody at room temperature (RT) for 1 h. Nuclei were stained with DAPI for 20 min at RT. Samples were mounted using Fluoromount-G (SouthernBiotech) and images were acquired using a Zeiss LSM700 confocal microscope (10× NA 0.45, 20× NA 0.8 objective). Quantifications were done using ImageJ software.

### VEGF Tail-Vein Injection and Immunoblotting

Hearts were collected from mice at 1, 2, 5 min after injection of PBS, or after 1, 2, 5, 10, 15, or 60 min after injection of VEGFA164 (canine, produced in insect cells; kind gift of Dr. Kurt Ballmer-Hofer, 250 μg per kg mouse) in the tail vein, snap-frozen in dry-ice / isopentane, and then stored at −80°C before use. Hearts were lysed in commercial RIPA buffer (ProteinSimple), total lysate was mixed with 2× SDS sample buffer, heated at 70°C for 10 min, loaded on NuPAGE Novex 4–12% Bis-Tris gels (Invitrogen) and processed for immunoblotting.

### Blood Component Count

Peripheral leukocyte counts in mice were determined at the governmental agency Statens Veterinärmedicinska Anstalt (Uppsala, Sweden) using “Quantifying Buffy Coat” (QBC-V) (Holst et al., 1994).

### RNA Sequencing

RNA was extracted from lungs of 5–8-week old wild-type and *Vegfr2*^*Y*949*F*/*Y*949*F*^ mice using the Qiagen RNeasy kit. mRNA purification was performed using Dynabeads mRNA Purification Kit (Ambion, Life Technologies AS, Oslo, Norway). mRNA libraries, three biological replicates per sample, were prepared using the Ion Total RNA-Seq Kit v2 (Life Technologies) using the standard protocol, and sequencing was performed on Ion Proton sequencer (Life Technologies). See Li et al. (2016) for further details on bulk RNAseq. Data were deposited with ArrayExpress, accession E-MTAB-9163.

### Statistical Analysis

Data are shown as means ± SEM. Statistical analyses were performed using nonparametric Mann–Whitney test to compare two unpaired groups and Wilcoxon test to compare two paired groups. Chi-squared test was used for survival assay. *p*-value < 0.05 was considered statistically significant. All statistical analyses were performed using GraphPad Prism Software.

## Results

### Outcome After MI in the *Vegfr2*^*Y*949*F*/*Y*949*F*^ Knock-in Mouse

**FIGURE 1:**
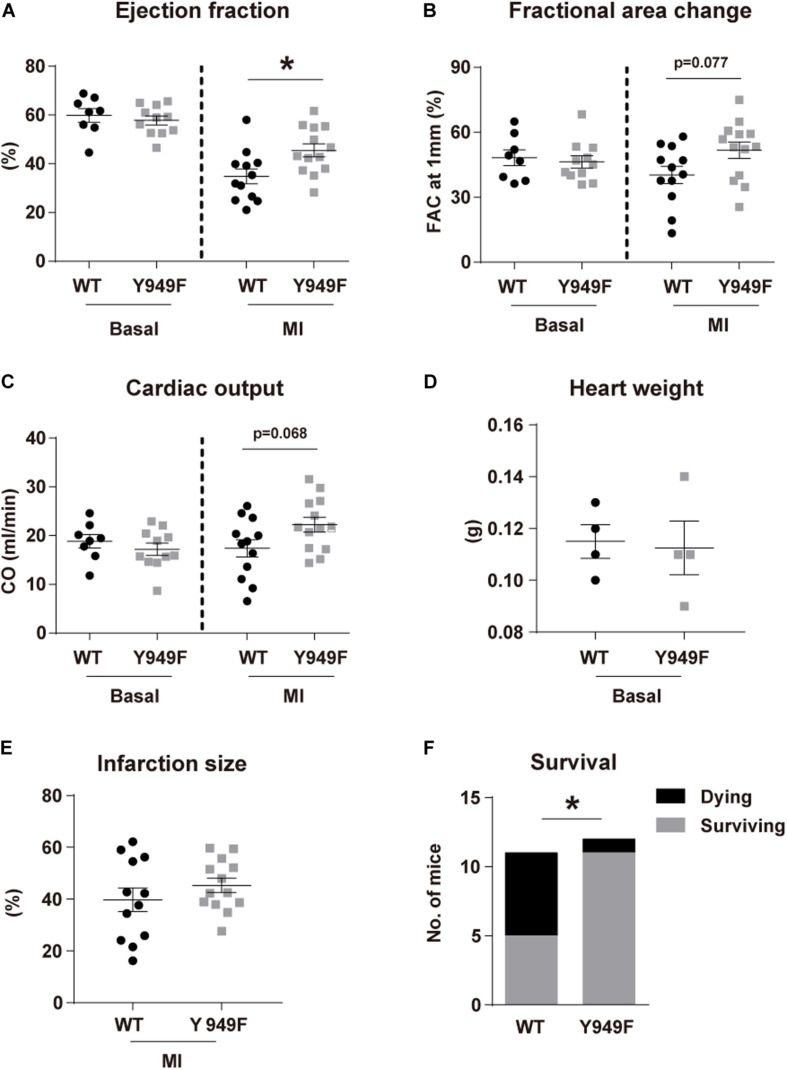
Improved cardiac function following MI in *Vegfr2*^*Y*949*F*/*Y*949*F*^ mice. **(A–C)** Heart function assessed using echocardiography; **(A)** ejection fraction, **(B)** fractional area change (FAC), **(C)** cardiac output (CO) in *Vegfr2*^*Y*949*F*/*Y*949*F*^ mice and WT littermates in baseline condition (*n* = 8–11 mice/strain), and at 24 h following myocardial infarction (*n* = 12–13 mice/strain, conducted at three different occasions). **(D,E)**. Heart weights before infarction (**D**; *n* = 4 mice/strain) and induced infarct size at 24 h after ligation **(E)** (*n* = 12–13 mice/strain) in WT and *Vegfr2*^*Y*949*F*/*Y*949*F*^ mice. Data in **(A–E)** are shown as mean ± SEM. Statistical significance was determined using Mann–Whitney test, *p < 0.05. **(F)** Survival after large infarct (*n* = 11–12/strain). Chi-squared test; *p < 0.05. For all panels, *n* refers to biological replicates.

The VEGFR2 Y949 phosphorylation site been implicated in regulation of the vascular barrier in different organs and in diseases such as cancer and retinopathy (Li et al., 2016; Smith et al., 2020). The potential impact of the pY949 pathway on the coronary circulation and on cardiac function has not been explored. We used mice in which VEGFR2 Y949 is replaced by F949 (*Vegfr2*^*Y*949*F*/*Y*949*F*^), and where adherens junctions in different vascular beds remain stable when exposed to VEGFA (Li et al., 2016; Smith et al., 2020). Under baseline conditions, *Vegfr2*^*Y*949*F*/*Y*949*F*^ mice displayed no differences in cardiac function compared with wild-type mice, as evaluated by echocardiography to determine EF, fractional area change (FAC) and cardiac output (CO) (Figures 1A–C). Next, mice were challenged by permanent LAD ligation and analyzed by echocardiography at 24 h after infarction. As shown in Figures 1A–C, the *Vegfr2*^*Y*949*F*/*Y*949*F*^ strain showed a trend to better performance with regard to FAC and CO, and displayed a significant better outcome in EF, compared to wild-type, after infarction. These data indicate that *Vegfr2*^*Y*949*F*/*Y*949*F*^ mice have a normal heart function under baseline conditions, and that they are more resilient when exposed to infarction. There was no difference in heart weights before challenge (Figure 1D) and the size of the induced infarcts (Figure 1E) were not different between strains. In accordance with the improved cardiac performance in mutant mice observed upon induction of smaller infarcts (Figures 1A–C), challenge of mice with large infarcts led to significantly better survival for the *Vegfr2*^*Y*949*F*/*Y*949*F*^ mice (11/12 survived) than for wild-type litter mates (6/11 survived; Figure 1F).

### Edema and Vascular Leakage Is Reduced in the *Vegfr2*^*Y*949*F*/*Y*949*F*^ Myocardium

**FIGURE 2:**
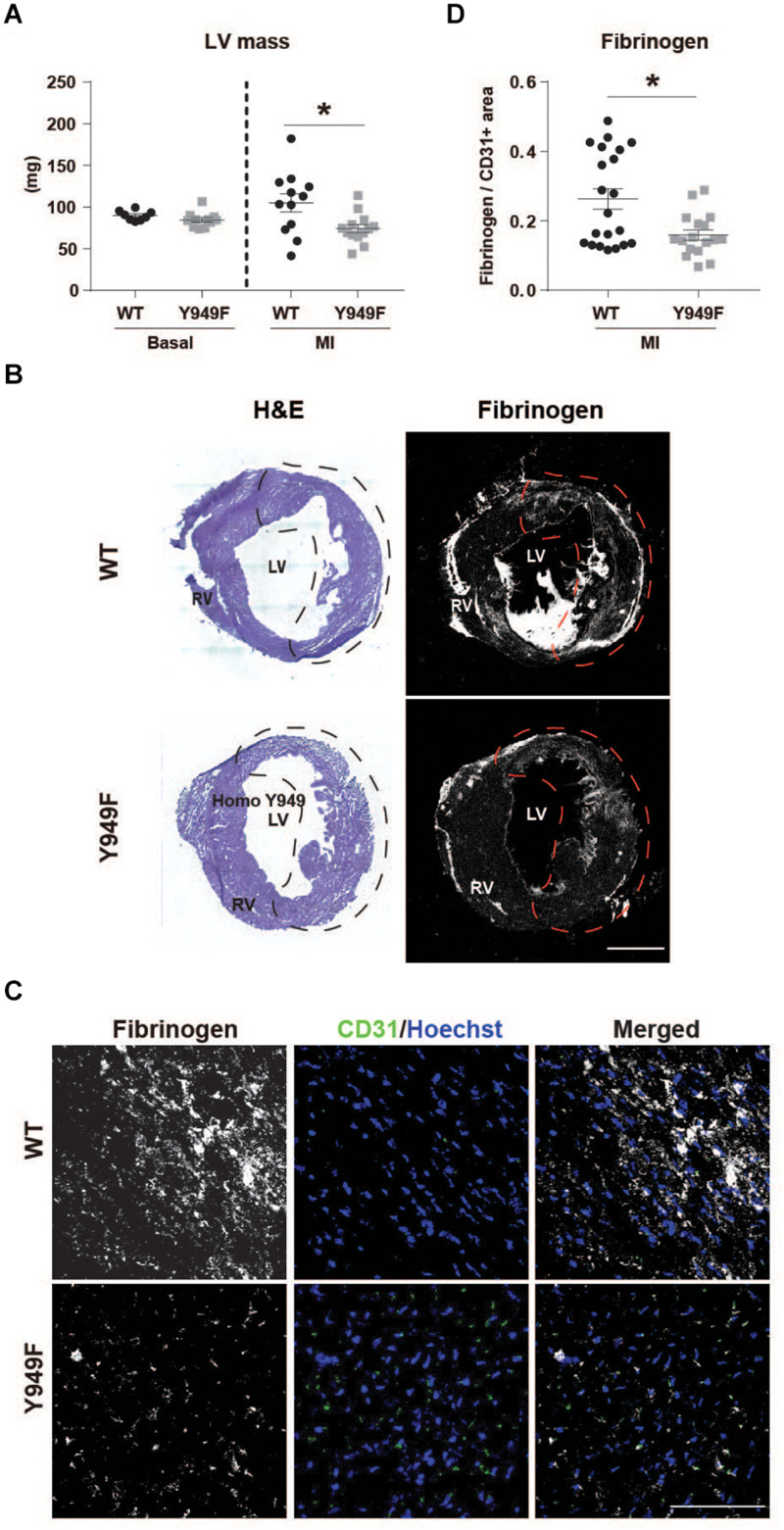
Reduced edema and fibrinogen leakage in *Vegfr2*^*Y*949*F*/*Y*949*F*^ MI hearts. **(A)** Left ventricular (LV) wall mass (mg) in WT and *Vegfr2*^*Y*949*F*/*Y*949*F*^ hearts at baseline condition (*n* = 8–11 mice/strain) and 24 h after MI (*n* = 12–13 mice/strain). Mann–Whitney test, *p < 0.05. **(B)** Hematoxylin/Eosin (H&E) and fibrinogen immunofluorescent staining on total WT (top) or Y949F (bottom) heart sections. Scale bar, 1500 μm. The infarct area detected using echocardiography, is indicated by stippled lines in black or red. **(C)** Fibrinogen (gray) and CD31 (green) immunofluorescent staining of WT and *Vegfr2*^*Y*949*F*/*Y*949*F*^ hearts at 3 days after MI; sections from the infarcted area. Scale bar, 100 μm. Hoechst (blue) shows nuclei. **(D)** Quantification of fibrinogen area normalized to CD31 area in randomly selected regions in total WT and *Vegfr2*^*Y*949*F*/*Y*949*F*^ hearts at 3 days after MI (*n* = 17–21 fields/section from 6 to 7 individual hearts/strain and 2–3 sections/heart). Mann–Whitney test, *p < 0.05. For all panels, *n* refers to biological replicates.

We have shown that VEGFR2 pY949-dependent signaling results in increased vascular permeability in the skin and trachea and that the permeability increase is not seen in the *Vegfr2*^*Y*949*F*/*Y*949*F*^ (Li et al., 2016). An underlying mechanism contributing to better cardiac function in mice lacking the pY949 signaling pathway could therefore involve improved vascular integrity after MI. In agreement, the left ventricular wall mass was significantly lower 24 h post MI in the *Vegfr2*^*Y*949*F*/*Y*949*F*^ mice compared to the wild-type (Figure 2A), indicating reduced edema compared to wild-type. Fibrinogen is a plasma protein which does not become deposited in tissues unless the vascular barrier is impaired (Davalos et al., 2012; Halder and Milner, 2020). Fibrinogen immunostaining normalized to vascular area was lower in the *Vegfr2*^*Y*949*F*/*Y*949*F*^ than in the wild-type mouse infarct area (Figures 2B,C; quantified in Figure 2D), indicating reduced macromolecular leakage, from the mutant vasculature.

**FIGURE 3:**
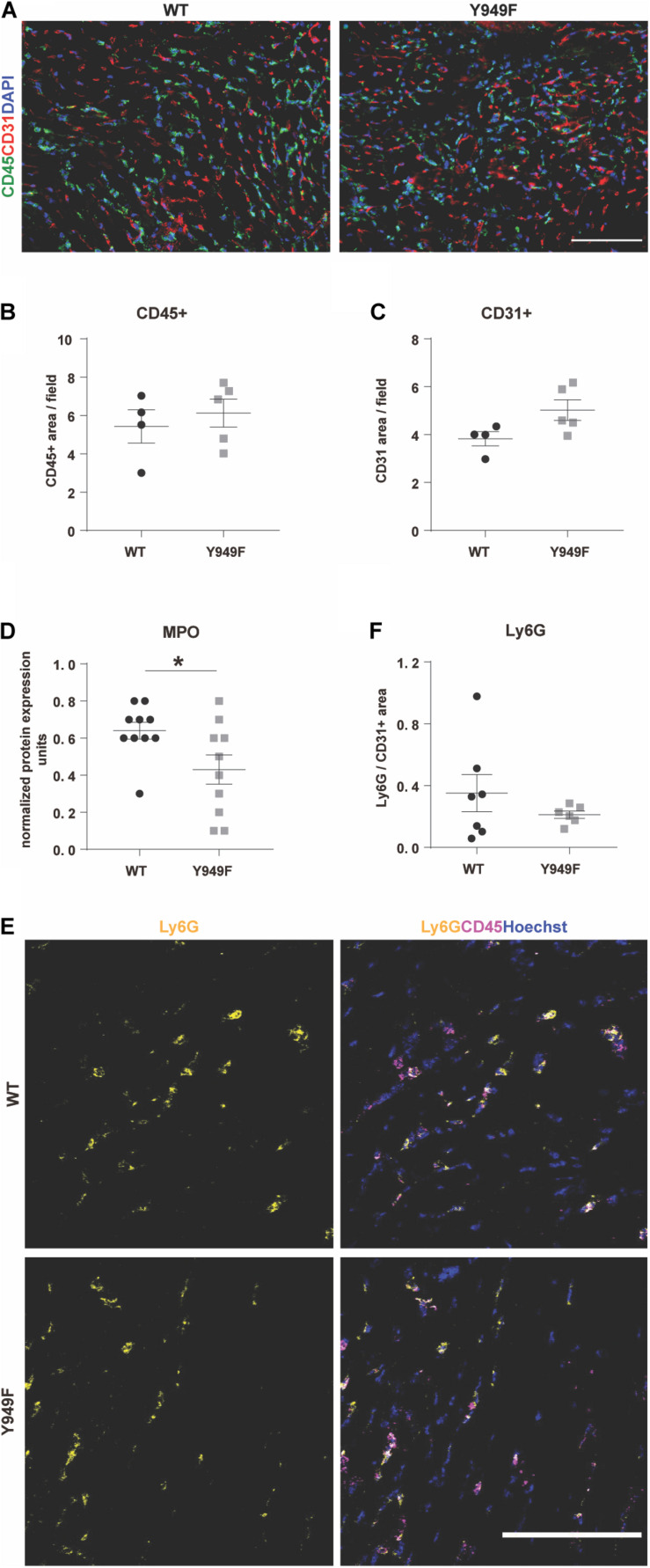
Inflammation, neutrophil infiltration and MPO levels in conjunction with MI. **(A)** Immunostaining for CD45 (green) and CD31 (red) to visualize infiltrating leukocytes and the vasculature, respectively, in the infarcted areas of WT and *Vegfr2*^*Y*949*F*/*Y*949*F*^ hearts at 3 days post-MI. DAPI (blue) shows nuclei. Scale bar, 100 μm. **(B,C)** Quantification of CD45-positive and CD31-positive area/field (*n* = 4–5 individual hearts/strain, 5 fields/section, and 3 sections/heart) in the infarcted part of the heart shows no significant differences using Mann–Whitney test. **(D)** Myeloperoxidase (MPO) protein levels in heart lysate measured using a multiplex inflammatory protein biomarker panel (*n* = 10 individual heart lysates/strain). Mann–Whitney test *p < 0.05. **(E)** Immunostaining for Ly6G (yellow) and CD45 (magenta) in the infarcted area of WT and *Vegfr2*^*Y*949*F*/*Y*949*F*^ hearts at 3 days after MI. Hoechst (blue) shows nuclei. Scale bar, 100 μm. **(F)** Quantification of Ly6G area normalized to CD31 area in the infarcted part of the heart (*n* = 6–7 individual hearts/strain, 5 fields/section, and 3 sections/heart) shows no significant differences using Mann–Whitney test. For all panels, *n* refers to biological replicates.

In contrast, vascular density or the density of CD45-positive inflammatory cells were similar in the infarct zone between wild-type and mutant hearts (Figures 3A–C).

### Neutrophil Infiltration Persistently Low in *Vegfr2*^*Y*949*F*/*Y*949*F*^ MI Tissue

Using a multiplex antibody panel to detect inflammatory markers in infarcted heart lysates, we noted the significantly lower expression of myeloperoxidase (MPO) in *Vegfr2*^*Y*949*F*/*Y*949*F*^ heart lysates compared to wild-type (Figure 3D; see Table 1 for inflammatory markers detected in the heart lysates). Elevated MPO levels in circulation are associated with inflammation and increased oxidative stress (Ndrepepa, 2019). An important potential source for MPO would be infiltrating neutrophils (Aratani, 2018). Neutrophils were detected in the infarcted areas using immunostaining for Ly6G. Most of the Ly6G-positive cells were also positive for CD45. Wild-type hearts showed large variability of infiltrating neutrophils, while the levels were consistently low in the mutant MI tissue (Figures 3E,F). The consistent low degree of infiltration of neutrophils in the Y949F MI tissue was not due to reduced neutrophil numbers in the unchallenged condition (Table 2). Indeed, there were no significant differences in the numbers of red blood cells, neutrophils, immune cells or monocytes between wild-type and *Vegfr2*^*Y*949*F*/*Y*949*F*^ in peripheral blood. Moreover, the reduced levels of MPO in infarcted heart lysates was not due to reduced basal expression of MPO. In fact, in the mutant, *Mpo* transcript levels were slightly elevated (0.142 DESeqlog2 increase according to statistical analysis of transcript expression, performed in R) in lung tissue from *Vegfr2*^*Y*949*F*/*Y*949*F*^ compared to wild-type (Li et al., 2016). Thus, these data show that base line levels of MPO expression and of circulating neutrophils were similar in the mutant and wild-type strains. Therefore, we suggest that the lower MPO levels detected in the mutant heart lysates was due to reduced infiltration of neutrophils as a consequence of the improved vascular barrier in the mutant *Vegfr2*^*Y*949*F*/*Y*949*F*^ strain and not e.g., to congenital changes in MPO expression or neutrophil numbers.

**TABLE 1:** Expression of inflammatory markers estimated from Proseek Multiplex CVDI assay on heart lysates after myocardial infarction.

Marker	Abbreviation	Levels^§^ in wild-type hearts^#^	Levels in *VEGFR2*^*Y*949*F*/*Y*949*F*^ *hearts*	*p*-value*
CD40 ligand	CD40L	2.920 ± 0.07	2.959 ± 0.05	0.601
Galectin-3	Gal-3	2.061 ± 0.09	1.942 ± 0.11	0.280
Myeloperoxidase	MPO	0.641 ± 0.04	0.420 ± 0.08	0.017*
β-nerve growth factor	β-NGF	1.832 ± 0.05	2.103 ± 0.35	0.280
Myoglobin	MB	5.216 ± 0.04	5.137 ± 0.04	0.190
Cathepsin D	CTSD	3.677 ± 0.04	3.617 ± 0.05	0.342
Melusin	ITGB1BP2	5.015 ± 0.11	4.994 ± 0.010	0.911
Dickkopf-related protein 1	Dkk-1	0.378 ± 0.05	0.387 ± 0.05	0.962
Heparin-binding EGF-like growth factor	HB-EGF	2.407 ± 0.04	2.422 ± 0.06	0.740
Fatty acid-binding protein, adipocyte	FABP4	2.268 ± 0.053	2.281 ± 0.08	0.684
Follistatin	FS	1.822 ± 0.070	1.695 ± 0.06	0.248

^#^ n = 10 heart lysates for each strain, i.e., 10 individual mice/strain. ^§^ Protein levels are shown as mean ± SEM and are given in arbitrary units. * p-values < 0.05 were considered significant; Mann–Whitney test.

**TABLE 2:** Peripheral blood cell counts.

Blood cell types	Wild-type^#^	*VEGFR2^*Y*949*F/Y*949*F*^*	*p*-Value*
Red blood cells (×10^12^/L)	8.72 ± 0.471^§^	8.85 ± 0.126	0.563
White blood cells (×10^9^/L)	3.32 ± 0.739	3.95 ± 0.780	0.329
∘ Neutrophils (×10^9^/L)	0.28 ± 0.040	0.40 ± 0.115	0.102
∘ Lymphocytes (×10^9^/L)	2.86 ± 0.689	3.26 ± 0.730	0.307
∘ Monocytes (×10^9^/L)	0.08 ± 0.040	0.12 ± 0.069	0.636

^#^ n = 5–6 for wild-type and mutant strains, respectively. ^§^ Values are shown as mean ± SEM. *There were no significant differences between groups using Mann–Whitney test.

### Reduced VE-Cadherin pY685 Levels in the *Vegfr2*^*Y*949*F*/*Y*949*F*^ Vasculature

**FIGURE 4:**
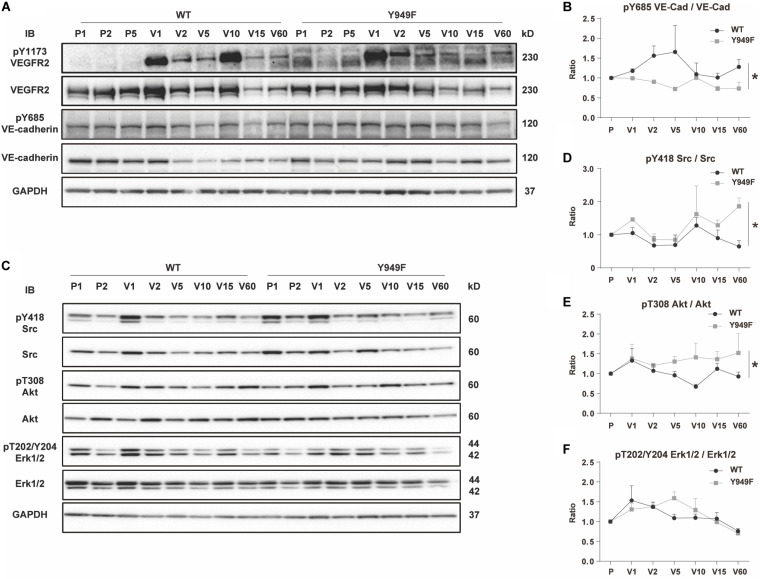
VEGF-induced VE-cadherin pY685 accumulation in hearts. **(A)** Immunoblot for pY1173 VEGFR2 and total VEGFR2, and pY685 VE-cadherin and total VE-cadherin on heart lysates from WT and *Vegfr2*^*Y*949*F*/*Y*949*F*^ mice. Mice were injected with PBS or VEGFA in the tail-vein, followed by circulation for different time periods; PBS was injected and left to circulate for 1, 2 or 5 min (denoted P1, P2, P5). Alternatively VEGFA was injected and left to circulate for 1, 2, 5 min etc. (denoted V1, V2, V5 etc.). Heart lysates were immunoblotted for 3-phosphate dehydrogenase (GAPDH) as a loading control. **(B)** Quantification of pY685 VE-cadherin normalized to total VE-cadherin in samples shown in **(A)**. **(C)** Immunoblotting for pY418 c-Src and total c-Src, pT308 Akt and total Akt, pT202/Y204 Erk1/2 and total Erk1/2. **(D–F)** Quantification of phosphorylated protein bands normalized to the corresponding total protein. *n* = 3; i.e., three independent replicates of experiments as shown in panel **(A)** with one mouse/time point for each strain and nine mice in total/strain for each experiment. Wilcoxon test, *p < 0.05.

To address the mechanisms underlying the vascular barrier properties in the *Vegfr2*^*Y*949*F*/*Y*949*F*^ vasculature, VEGFA, or as a control, PBS, was injected in the tail-vein of wild-type and mutant mice, followed by harvesting of hearts after different time periods of circulation. Hearts were lysed and samples processed for immunoblotting. Phosphorylation on Y685 in VE-cadherin correlates with vascular permeability (Orsenigo et al., 2012; Wessel et al., 2014; Smith et al., 2020). Levels of pY685 in VE-cadherin in the heart endothelium increased at 2–5 min after VEGFA injection in the wild-type, whereafter it returned to basal levels. In contrast, VE-cadherin phosphorylation remained unaffected in the *Vegfr2*^*Y*949*F*/*Y*949*F*^ heart endothelium (Figures 4A,B). To ensure that VEGFR2 had been activated, samples were probed also with antibodies against pY1173 VEGFR2; the main phosphorylation sites in VEGFR2. As seen in Figure 4A, VEGFR2 became phosphorylated in both wild-type and mutant hearts in response to VEGFA. Moreover, downstream signaling pathways involving activation of c-Src, Akt, and Erk1/2 were examined using phosphor-specific antibodies. As seen in Figures 4C–F, pY418 c-Src, pT308 Akt, and pT202/Y204 Erk1/2 were induced to wild-type levels or even elevated levels in the *Vegfr2*^*Y*949*F*/*Y*949*F*^ endothelium, further demonstrating the capacity of the mutant receptor to respond to VEGFA. The elevated responsiveness of the mutant receptor may indicate the establishment of compensatory mechanisms in the mutant strain.

## Discussion

CVD, mainly MI, remains a major cause of premature death globally. Metabolic diseases such as obesity and diabetes predispose to CVD and while women and young adults previously were spared, the spread of these metabolic diseases combined with sedentary life style patterns now result in broader sections of the population being affected (Low Wang et al., 2016). Treatment of the acute infarction includes coronary angioplasty and stenting, and bypass surgery. A major challenge to those who survive the acute phase, is to restore and retain myocardial function by minimizing myocardial death due to tissue damage. The coronary circulation is vital to maintain optimal cardiomyocyte function (Hausenloy et al., 2019).

**FIGURE 5:**
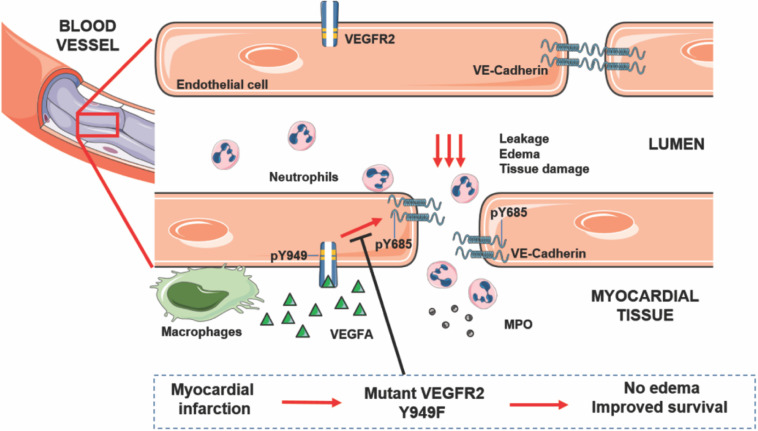
VEGFA/VEGFR2 regulates edema and tissue damage in myocardial infarction. Activation of VEGFR2 on endothelial cells leads to phosphorylation on Y949 and subsequently, phosphorylation of VE-cadherin on Y685, leading to dismantling of adherens junctions and leakage, edema, neutrophil infiltration, MPO release, and tissue damage. Mutation of Y949 in VEGFR2 halts edema and improves survival after myocardial infarction.

The hypothesis underlying the current study was that alleviation of edema in conjunction with MI would decrease tissue damage and allow improved cardiac function, of vital consequence for the long-term outcome after MI. In accordance, we show that genetic interference with the ability of VEGFA/VEGFR2 to mediate increased vascular permeability, correlated with reduced left ventricular edema in mice and improved survival after MI, while coronary vascular density was unaffected (see Figure 5 for a schematic outline). The MI-challenged mutant *Vegfr2*^*Y*949*F*/*Y*949*F*^ mice showed better cardiac function with a significant increase in EF, compared to the wild-type littermates. The principal underlying mechanism involved persistent stability of adherens junctions in the presence of VEGFA, as indicated by the reduced phosphorylation of VE-cadherin on Y685 in *Vegfr2*^*Y*949*F*/*Y*949*F*^ heart ECs in response to VEGFA.

Phosphorylation of VE-cadherin on Y685, correlates with adherens junction disruption and vascular permeability (Wessel et al., 2014). Orsenigo et al. (2012) showed that flow-dependent phosphorylation of Y685 in VE-cadherin occurs in venules and not in arteries, which is agreement with that VEGFA induces permeability in prevenular capillaries and venules but not in arteries (Honkura et al., 2018). c-Src is implicated as the tyrosine kinase responsible for phosphorylation of VE-cadherin. Thus, genetic inactivation or pharmacological suppression of c-Src activity stabilizes endothelial adherens junctions (Weis et al., 2004). In heart lysates, c-Src activity was induced to the same extent or higher in the *Vegfr2*^*Y*949*F*/*Y*949*F*^ mutant compared to wild-type in response to VEGFA (Figure 4), and therefore, we cannot infer from this study that c-Src is responsible for VE-cadherin phosphorylation in the coronary vasculature. However, constitutive *c-Src* gene inactivation is accompanied by reduced edema and improved long-term outcome after MI (Weis et al., 2004) strongly indicating that c-Src indeed phosphorylates VE-cadherin. A caveat in analysis of c-Src activation is the lack of reagents that specifically detects only activated c-Src and not the related Fyn and Yes, thus, it is possible that reduced levels of c-Src activity in the mutant was compensated for by increased Fyn/Yes activities. Moreover, it is known that barrier regulation involves translocation of c-Src to endothelial junctions which can be studied in tissue culture or in whole-mounted tissues (Sun et al., 2012; Li et al., 2016). As the coronary vasculature does not lend itself to whole-mount procedures, the subcellular localization of pY418 c-Src in the coronary endothelium could not be determined.

A limitation in the VE-cadherin analyses performed here, is the use of total organ lysates rather than e.g., isolated ECs. However, isolation of ECs after *in situ* VEGFA stimulation would introduce technical issues in maintaining the phosphorylation patterns over the time-consuming isolation procedure. An alternative strategy would be to treat isolated cardiac ECs with VEGFA followed by biochemical analyses. The drawback with this procedure is that the establishment of cells in culture attenuates the organotypic features of the cells. We conclude that, as both VEGFR2 and VE-cadherin are preferentially expressed on ECs and not for example on cardiomyocytes, the analyses performed in this study can be assumed to reflect endothelial-specific regulation of vascular permeability in the heart. Moreover, since the coronary vasculature needs to be studied by sectioning the tissue the degree of stability of the endothelial cell–cell contacts cannot be visualized as for tissues that can be whole-mounted (skin, trachea, mesentery, diaphragm). However, we suggest that based on the reduced LV edema, the reduced fibrinogen/fibrin deposition and the changes in MPO levels and low neutrophil accumulation in the heart, combined with the reduced VE-cadherin phosphorylation on Y685, strongly indicate that the stringency of the vascular barrier is maintained in the mutant tissues including the heart, when exposed to VEGFA. In contrast, exposure to VEGFA leads to barrier breakdown in the wild-type tissues.

The data presented here complement and advance previous studies showing that VEGFA-induced phosphorylation of VE-cadherin is reduced in *Vegfr2*^*Y*949*F*/*Y*949*F*^ lung ECs, correlating with reduced vascular leakage (Li et al., 2016). We now confirm this pattern in the heart, thus, VEGFA/VEGFR2 regulation of vascular permeability in the coronary vasculature appears to be similar to that in several other organs such as the brain, the pancreas, the heart and the skin. Moreover, as the vascular development, vascular density and morphology for example in the kidney and retina, was unaffected in the *Vegfr2*^*Y*949*F*/*Y*949*F*^ model (Li et al., 2016), it appears that limiting VEGFA-regulated permeability is dispensable for physiological angiogenesis and inflammation. Of note, infiltration of CD45+ cells was unaffected in the VEGFR2 mutant hearts (cf. Figure 3). In agreement, inflammatory cell infiltration in melanoma occurs to the same extent when comparing *Vegfr2*^*Y*949*F*/*Y*949*F*^ and wild-type mice (Li et al., 2016) and similarly, inflammation accompanying oxygen-induced retinopathy is unaffected by the VEGFR2 mutation (Smith et al., 2020). A fraction of the CD45+ cells infiltration the myocardial tissue after infarction were neutrophils (cf. Figure 3), which are highly motile cells. The spread of neutrophil infiltration between individuals was much higher in the wild-type hearts than in the *Vegfr2*^*Y*949*F*/*Y*949*F*^ hearts, which may be a consequence of the more extensive edema in the wild-type infarcted hearts (cf. Figure 2), correlating with the increased demise of wild-type mice (cf. Figure 1), compared to the mutants. However, we cannot exclude that the extent of infiltration of subsets of inflammatory cell populations into the infarcted area was affected in the mutant at earlier or later time points after infarction.

We conclude that suppressing vascular permeability in the acute MI phase while preserving other aspects of VEGFR2 functions required e.g., for subsequent formation of collaterals is a highly relevant option for future refined MI treatment.

## Data Availability Statement

The RNAseq data was deposited with ArrayExpress: accession E-MTAB-9163. The raw data supporting the conclusions of this article will be made available by the authors, without undue reservation.

## Ethics Statement

The animal study was reviewed and approved by Uppsala University (approval reference number 5.8.18-06789/2018) and Göteborg University Animal Ethics Committees.

## Author Contributions

XL and LC-W devised the project and the main conceptual ideas, and composed the manuscript. BR, MS, and ML performed the cardiovascular tests. XL, MS-J, SS, NP, and PM performed the biochemical and cell biological assays. XL, MS-J and ML performed the data analyses. All authors commented and approved the text.

## Conflict of Interest

The authors declare that the research was conducted in the absence of any commercial or financial relationships that could be construed as a potential conflict of interest.
